# Association between Serum Growth Hormone Levels and Nonalcoholic Fatty Liver Disease: A Cross-Sectional Study

**DOI:** 10.1371/journal.pone.0044136

**Published:** 2012-08-31

**Authors:** Lei Xu, Chengfu Xu, Chaohui Yu, Min Miao, Xuequn Zhang, Zhongwei Zhu, Xiaoyun Ding, Youming Li

**Affiliations:** 1 Department of Gastroenterology, Ningbo No. 1 Hospital, Ningbo, China; 2 Department of Gastroenterology, the First Affiliated Hospital, College of Medicine, Zhejiang University, Hangzhou, China; 3 Department of Gastroenterology, Zhenhai Lianhua Hospital, Ningbo, China; University of Cordoba, Spain

## Abstract

Growth hormone (GH) is an important regulator of metabolism and body composition. GH deficiency is associated with increased visceral body fat and other features of the metabolic syndrome. Here we performed a cross-sectional study to explore the association of GH levels with nonalcoholic fatty liver disease (NAFLD), which is considered to be the hepatic manifestation of the metabolic syndrome. A total of 1,667 subjects were diagnosed as NAFLD according the diagnostic criteria, and 5,479 subjects were defined as the controls. The subjects with NAFLD had significantly lower levels of serum GH than the controls. Those with low GH levels had a higher prevalence of NAFLD and the metabolic syndrome. A stepwise logistic regression analysis showed that GH levels were significantly associated with the risk factor for NAFLD (OR = 0.651, 95%CI = 0.574–0.738, *P*<0.001). Our results showed a significant association between lower serum GH levels and NAFLD.

## Introduction

Nonalcoholic fatty liver disease (NAFLD) refers to a wide spectrum of liver diseases, ranging from simple steatosis through nonalcoholic steatohepatitis (NASH) to cirrhosis. NAFLD is closely associated with features of the metabolic syndrome, including obesity, type 2 diabetes, hypertension, and dyslipidemia. NAFLD is therefore considered as the hepatic manifestation of the metabolic syndrome [1], [2].

Growth hormone (GH) originates from the anterior pituitary gland and is an important regulator of nutritional metabolism and body composition. Low GH secretion from the pituitary is associated with increased visceral body fat and other features of the metabolic syndrome. For example, in a previous study, patients with GH deficiency had significance higher body mass index (BMI) and waist circumference, and elevated serum total cholesterol, low-density lipoprotein cholesterol and triglycerides than the controls [3]. Adult patients with GH deficiency also had a higher prevalence of the metabolic syndrome than the controls [4], [5]. GH therapy decreased the prevalence of the metabolic syndrome and improved metabolic status [6], [7].

Some studies have noted the high prevalence of NAFLD in GH deficiency. Ichikawa *et al.* [8] reported that in patients with adult onset anterior lobe pituitary hormone deficiency (with or without GH deficiency), NAFLD was more frequently observed in patients with GH deficiency. Decreased GH levels were also associated with the severity of hepatic steatosis in other studies [3], [9]. Furthermore, hypothalamic/pituitary dysfunction may be accompanied by progressive NAFLD and excessive weight gain, impaired glucose tolerance, and dyslipidemia [10]. However, Gardner *et al.* [11] recently reported that there was no significant difference in the incidence of NAFLD between GH deficiency and the controls. The sample sizes of these studies were small and the study subjects were almost all patients with hypothalamic/pituitary illness.

So far, the association between GH levels and NAFLD among the general population remains unclear. The aim of this study was to investigate the association between the GH levels and NAFLD in a large population survey.

## Materials and Methods

### Ethics Statement

Before participation in the study and after all procedures had been explained, verbal informed consent was obtained from each subject enrolled. Verbal consent was recorded by the physician who explained the study procedures. Written informed consent was not required because of the observational nature of the investigation. The Ethics Committee of the First Affiliated Hospital, College of Medicine, Zhejiang University approved the study’s protocol and manner of consent.

### Study Design and Subjects

To evaluate the relationship between serum GH levels and NAFLD, a cross-sectional study was conducted among the employees of Zhenhai Refining & Chemical Company Ltd. (Ningbo, China). These employees were enrolled at the time of their annual health examination between March 1, 2009 and December 31, 2009. The majority of these subjects had taken part in our other studies [12]. The following individuals were excluded from the study, based on medical history and self-reported medication use: (1) those with excessive alcohol consumption (more than 140 g/week for men and 70 g/week for women) (n = 697); (2) those with a history of viral hepatitis, autoimmune hepatitis, or other liver disease (n = 797); and (3) those taking antihypertensive, antidiabetic agents, or lipid-lowering agents (n = 702). A total of 7146 subjects were enrolled (4622 men and 2524 women; mean age of 45.8±15.1 years and 49.4±13.2 years, respectively).

### Data Collection

All subjects underwent a clinical examination, which included a medical history and health habit inventory taken by a physician, anthropometric measurements, hepatic ultrasonic examination, and biochemical measurements. The examinations were administered in the morning. The subjects had been instructed to fast for at least 12 hours prior to the examination and to refrain from exercise during the day before their examination. Blood pressure was measured using an automated sphygmomanometer with the subject in a sitting position. Systolic blood pressure and diastolic blood pressure were measured at the first and fifth Korotkoff phases, respectively. Mean arterial pressure was calculated as diastolic blood pressure plus one-third pulse pressure. Pulse pressure was defined as the difference between systolic and diastolic blood pressure. Standing height and body weight were measured without shoes or outer clothing. BMI was calculated as weight in kilograms divided by height in meters squared (kg/m^2^). Waist circumference was measured with the measuring tape positioned midway between the lowest rib and the superior border of the iliac crest as the subject exhaled normally.

Fasting blood samples were obtained from an antecubital vein of each subject in the morning, and the samples were used for the analysis of biochemical values. The values included alanine aminotransferase (ALT), γ-glutamyltransferase (GGT), triglycerides, total cholesterol, high- and low-density lipoprotein cholesterol (HDL-C and LDL-C, respectively), and fasting blood glucose. All values were measured with an Olympus AU640 autoanalyzer (Olympus, Kobe, Japan) using standard methods. The serum GH levels were determined via a protein chip-chemiluminescence system (HealthDigit, Shanghai, China).

The diagnosis of hepatic steatosis was based on the liver ultrasound performed with a 3.5-MHz transducer (Nemio 20, Toshiba, Japan). A trained ultrasonographist who was blinded to the clinical and laboratory data carried out the ultrasound examination. Hepatic steatosis was diagnosed according to characteristic echo patterns, such as diffuse hyperechogenicity of the liver relative to the kidneys, ultrasound beam attenuation, and poor visualization of intrahepatic structures [13]. NAFLD was diagnosed after exclusion of alcoholic consumption, or viral or autoimmune liver disease [14], [15]. The diagnosis of the metabolic syndrome was based on the revision of the definition established by the National Cholesterol Education Program Adult Treatment Panel III, recommended by the Asia-Pacific Working Party on NAFLD 2006 [16].

### Statistical Analyses

Data are presented as the mean and standard deviation (SD) when normally distributed, or as the median if skewed. Differences between groups were analyzed with the unpaired Student’s *t*-test for normally distributed data or the Mann-Whitney *U* test for skewed data. The chi-squared (*χ* ^2^) test was used for comparisons of categorical variables. Pearson’s or Spearman’s analysis was used to determine correlations between parameters. A multiple stepwise regression analysis (backward: Wald; cutoff for entry: 0.05, for removal: 0.10) tested the risk factors for NAFLD. *P*<0.05 (2-tailed test) was considered statistically significant.

## Results

### Characteristics of Subjects

Of the 7146 individuals enrolled in the study, 1667 were diagnosed with NAFLD, and the remaining 5479 were considered the control group. The average age of the NAFLD group was older when compared to the controls (*P*<0.01). Most of the clinical and metabolic characteristics, including waist circumference, systolic and diastolic blood pressures, triglycerides, total cholesterol, HDL-C, LDL-C, and fasting blood glucose were more unfavorable in subjects with NAFLD when compared to the controls (all with *P*<0.01). Subjects with NAFLD also presented higher ALT and GGT levels than the controls. Meanwhile, serum GH levels were significantly lower in the NAFLD group (Table 1).

**Table 1:** The characteristics of the subjects with NAFLD and controls.

	NAFLD (n = 1667)	Controls (n = 5479)	*t* value	*P* value
Age (years)	49 (40–58)	44 (35–57)	9.598^a^	<0.001
Gender(male/female)	1213/454	3409/2070	62.224^b^	<0.001
BMI (kg/m^2^)	24.64±3.01	22.75±2.96	22.638	<0.001
WC (cm)	85 (80–90)	79 (72–85)	22.677 a	<0.001
SBP (mmHg)	129.3±17.5	124.1±16.9	11.071	<0.001
DBP (mmHg)	81.4±10.9	77.9±10.3	12.070	<0.001
ALT (U/L)	21 (15–33)	16 (12–23)	18.269^a^	<0.001
GGT ((U/L)	27 (19–43)	20 (14–30)	18.193^a^	<0.001
TG (mmol/L)	1.49 (1.02–2.11)	1.10 (0.79–1.57)	17.947^a^	<0.001
TC (mmol/L)	5.13±0.92	4.90±0.91	8.765	<0.001
HDL-C (mmol/L)	1.30 (1.15–1.47)	1.37 (1.19–1.61)	9.263^a^	<0.001
LDL-C (mmol/L)	3.00±0.75	2.86±0.73	7.186	<0.001
FBG (mmol/L)	4.89 (4.60–5.31)	4.76 (4.49–5.09)	10.171^a^	<0.001
GH (ng/mL)	0.02 (0.01–6.01)	0.11 (0.02–7.09)	13.909 a	<0.001

The data are expressed as the mean ± SD or median (IQR) depending on the data distribution.

a *z* value; ^b^ *χ* ^2^ value.

Abbreviations: ALT: alanine aminotransferase; BMI: body mass index; DBP: diastolic blood pressure; FBG: fasting blood glucose; GGT: γ-glutamyltransferase; GH: growth hormone; HDL-C: high-density lipoprotein cholesterol; LDL-C: low-density lipoprotein cholesterol; SBP: systolic blood pressure; TG: triglyceride; TC: total cholesterol; WC: waist circumference.

**Table 2:** Correlations between GH and features of the metabolic syndrome.

	WC	SBP	DBP	TG	HDL-C	FBG
*r* value	-0.222	-0.108	-0.077	-0.151	0.164	-0.028
*P* value	<0.001	<0.001	<0.001	<0.001	<0.001	0.018

Abbreviations: DBP: diastolic blood pressure; FBG: fasting blood glucose; GH: growth hormone; HDL-C: high-density lipoprotein cholesterol; SBP: systolic blood pressure; TG: triglyceride; WC: waist circumference.

**Table 3:** Stepwise logistic regression analysis using NAFLD as dependent variable.

Variables	β	SE	Wald χ^2^	*P*	OR	95% CI of OR
Age (years)	0.008	0.002	13.632	<0.001	1.008	1.004–1.013
Body mass index (kg/m^2^)	0.085	0.015	32.060	<0.001	1.089	1.057–1.121
Waist circumference (cm)	0.023	0.005	19.680	<0.001	1.024	1.013–1.034
Alanine aminotransferase (U/L)	0.009	0.002	26.152	<0.001	1.009	1.006–1.013
Total cholesterol (mmol/L)	0.150	0.04	14.122	<0.001	1.162	1.075–1.257
Triglyceride (mmol/L)	0.115	0.032	12.626	<0.001	1.122	1.053–1.196
HDL cholesterol (mmol/L)	-0.500	0.135	13.741	<0.001	0.606	0.465–0.790
Fasting plasma glucose (mmol/L)	0.099	0.031	10.090	0.001	1.104	1.039–1.173
Growth hormone (ng/mL)	-0.429	0.064	44.749	<0.001	0.651	0.574–0.738

Abbreviations: β, partial regression coefficient; SE, standard error of partial regression coefficient; OR, odds ratio; CI, confidence interval; HDL, high-density lipoprotein.

**Figure 1:**
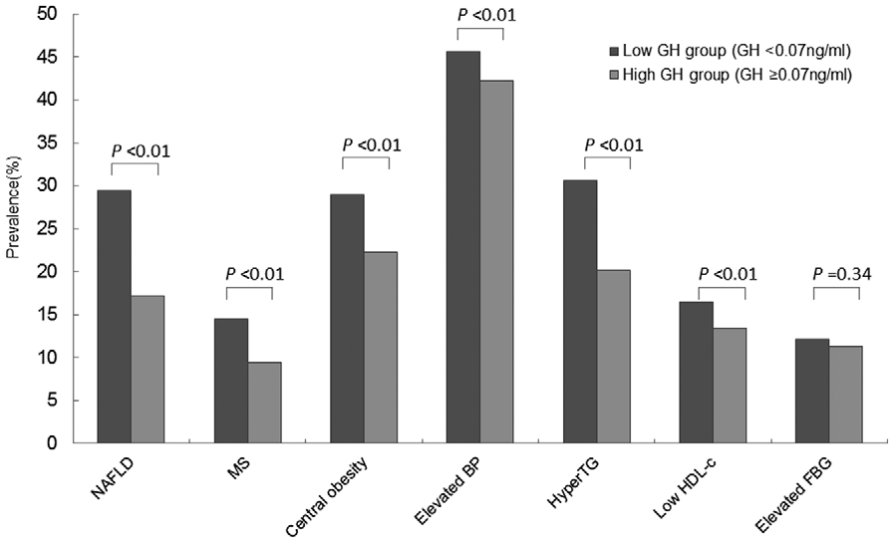
The prevalence of NAFLD and the metabolic syndrome in the subjects with different serum GH levels. The group with higher GH levels showed a lower prevalence of NAFLD, the metabolic syndrome, central obesity, elevated blood pressure, hypertriglyceridemia, and low HDL-C level than the group with lower GH levels. Abbreviations: BP: blood pressure; FBG: fasting blood glucose; GH: growth hormone; HDL-c: high-density lipoprotein cholesterol; MS: metabolic syndrome; NAFLD: nonalcoholic fatty liver disease; TG: triglyceride.

### Association between Serum GH Levels and NAFLD

GH levels showed an inverse correlation with the features of the metabolic syndrome [waist circumference (*r* = –0.222, *P*<0.001), systolic blood pressure (*r* = –0.108, *P*<0.001), diastolic blood pressure (*r* = –0.077, *P*<0.001), triglycerides (*r* = –0.151, *P*<0.001), fasting blood glucose (*r* = –0.028, *P*<0.018) and HDL-C (*r* = 0.164, *P*<0.001)] (Table 2). A stepwise multiple logistic regression was performed to investigate the relationship between the GH levels and NAFLD. Thirteen variables including age, gender, BMI, waist circumference, mean arterial pressure, ALT, GGT, total cholesterol, triglycerides, HDL-C, LDL-C, fasting plasma glucose, and GH were entered into the original equation. The results showed that seven variables, namely age, BMI, waist circumference, ALT, total cholesterol, triglycerides and fasting plasma glucose were significantly and positively associated with NAFLD; while HDL-C and GH were inversely correlated with NAFLD (Table 3).

To further analyze the relationship between NAFLD and GH levels, subjects with serum GH levels <0.07 ng/mL were defined as the lower GH group and those with serum GH levels ≥0.07 ng/mL were defined as the higher GH group (0.07 ng/mL was the median GH concentration). NAFLD was more prevalent in the lower GH group (28.8%), compared with the higher GH group (17.2%; *χ* ^2^ = 134.4, *P*<0.001). Similarly, the metabolic syndrome was more prevalent in the lower GH group (14.5%), compared with the higher GH group (9.5%; *χ* ^2^ = 40.9, *P*<0.001). In addition, the lower GH group had higher prevalence of metabolic syndrome features (Figure 1).

## Discussion

In the present cross-sectional study of 7146 individuals, serum GH levels were significantly associated with the prevalence of NAFLD and the metabolic syndrome. NAFLD subjects had lower GH levels, and the prevalence of NAFLD negatively correlated with GH levels. We also observed that GH levels were inversely correlated with the metabolic syndrome. Furthermore, the stepwise multiple logistic regression analysis showed a significant link between lower serum GH levels and the risk factor of NAFLD.

GH deficiency and the metabolic syndrome share many similar features, including abdominal/visceral obesity, insulin resistance, premature atherosclerosis, and increased mortality from cardiovascular diseases [17]. Adults with little or no detectable GH also often have a higher BMI, waist circumference, waist-to-hip ratio, fasting glucose, cholesterol, and other metabolic disorders [3]. NAFLD is the hepatic manifestation of the metabolic syndrome, and its higher prevalence among GH-deficient adults has already been noted [3], [8], [10]. These findings are in agreement with the results of the present study, in that the prevalence rate of NAFLD was higher in those subjects with GH levels <0.07 ng/mL than in those whose GH levels were higher. Meanwhile, NAFLD subjects also had lower GH levels in our study. This is consistent with the report of Fusco *et al.* [18], who found after growth-hormone-releasing hormone (GHRH) and arginine stimulation tests that NAFLD patients had lower peak levels of serum GH and insulin-like growth factor (IGF1), and higher levels of serum GH-binding protein (GHBP) and insulin-like growth factor-binding protein 3 (IGFBP3).

However, a recent small sample case-control study showed that GH deficiency patients did not have a higher incidence of hepatic steatosis compared to age-and BMI-matched controls [11]. In that study, proton magnetic resonance spectroscopy (^1^H-MRS) was used to diagnose hepatic steatosis, which is a more sensitive technique than computed tomography or ultrasonography. Therefore, further research is required to clarify the complex relationship between GH, hepatic steatosis, and the metabolic syndrome.

While the above has argued that the association between lower GH levels and NAFLD was due to the latter’s status as the metabolic syndrome component, it may be more probable that insulin resistance is the link between GH and NAFLD. Insulin resistance has a central role in the pathogenesis of NAFLD, and low GH level is also associated with insulin resistance. Insulin can directly suppress GH secretion from the pituitary [19], while more severe insulin resistance may impair GHRH and the arginine-induced GH response in NAFLD patients [20].

Furthermore, inflammation is characteristic of the pathological progression of NAFLD [21], and GH deficiency often leads to an inflammatory process; GH deficient adults were reported to have higher levels of inflammatory markers such as C-reactive protein and tumor necrosis factor [22], [23]. In addition, oxidative stress is another important feature of the pathogenesis of NAFLD [24], and lower GH levels often accompany oxidative stress. Increased levels of oxidative stress were observed in the serum and liver of a GH-deficient patient, it was decreased after GH-replacement therapy [25]. Other investigations also showed that GH had an antioxidant role [26], [27].

Regrettably, our present cross-sectional study did not establish a cause for the association between low GH levels and NAFLD. The study’s findings were also limited in that NAFLD was diagnosed solely by ultrasonographic methods, which cannot distinguish NASH from simple steatosis. Nevertheless, ultrasonography is widely used for population-based studies, with reasonable accuracy [28]. Furthermore, our initial study design did not allow for the examination of insulin levels and insulin resistance, although insulin resistance may be an important link between low GH levels and NAFLD. For the same reason, IGF-1 was not examined; this hampers our complete understanding of the association between NAFLD and the GH/IGF-1 axis. Moreover, the secretion of GH is pulsatile, a single measurement of serum GH level may not adequate to reflect true 24h GH levels. Finally, inflammatory markers such as interleukin-6 and tumor necrosis factor were not measured, which could provide important information regarding the association between GH levels and NAFLD.

In conclusion, in this cross-section study we found that low serum GH levels were significantly associated with NAFLD. This suggests that GH might play an important role in the diagnosis and physio-pathological process of NAFLD.
